# ADAMTS16 mutations sensitize ovarian cancer cells to platinum-based chemotherapy

**DOI:** 10.18632/oncotarget.11120

**Published:** 2016-08-08

**Authors:** Maya Yasukawa, Yuexin Liu, Limei Hu, David Cogdell, Kshipra M. Gharpure, Sunila Pradeep, Archana S. Nagaraja, Anil K. Sood, Wei Zhang

**Affiliations:** ^1^ Departments of Pathology, The University of Texas MD Anderson Cancer Center, Houston, Texas, USA; ^2^ Gynecologic Oncology and Reproductive Medicine, The University of Texas MD Anderson Cancer Center, Houston, Texas, USA; ^3^ Cancer Biology, The University of Texas MD Anderson Cancer Center, Houston, Texas, USA; ^4^ Center for RNA Interference and Non-Coding RNA, The University of Texas MD Anderson Cancer Center, Houston, Texas, USA; ^5^ Department of Obstetrics and Gynecology, Showa University School of Medicine, Shinagawa-ku, Tokyo, Japan; ^6^ Department of Cancer Biology, Comprehensive Cancer Center of Wake Forest Baptist Medical Center, Winston-Salem, North Carolina, USA; ^7^ Bioinformatics and Computational Biology, The University of Texas MD Anderson Cancer Center, Houston, Texas, USA

**Keywords:** ovarian cancer, ADAMTS16, chemosensitivity, platinum-based chemotherapy, BRCAness

## Abstract

Ovarian cancer is one of the most lethal malignant tumors in women. The prognosis of ovarian cancer patients depends, in part, on their response to platinum-based chemotherapy. Our recent analysis of genomics and clinical data from the Cancer Genome Atlas demonstrated that somatic mutations of ADAMTS 1, 6, 8, 9, 15, 16, 18 and L1 genes were associated with higher sensitivity to platinum and longer progression-free survival, overall survival, and platinum-free survival duration in 512 patients with high-grade serous ovarian carcinoma. Among the *ADAMTS* mutations*, ADAMTS16* is the most commonly affected gene in ovarian cancer. However, the functional role of these mutations in ovarian cancer cells is largely unknown. We performed *in vitro* studies to compare the functional effects of the six identified ADAMTS missense mutations on the platinum sensitivity of ovarian cancer cells. We also used a well-characterized *in vivo* mouse model to evaluate the response of ovarian cancer cells with *ADAMTS16* mutations to platinum-based therapy. Our results showed that exogenously expressed *ADAMTS16* missense mutations inhibited cell growth or sensitized tumor cells to cisplatin and inhibited tumor growth *in vivo*. Orthotopic xenograft experiments showed that mice injected with ovarian cancer cells that exogenously expressed *ADAMTS16* mutations had a better response to cisplatin treatment. Thus, these functional studies provide evidence that mutations of *ADAMTS16* actively contribute to therapeutic response in ovarian cancer.

## INTRODUCTION

High-grade serous ovarian cancer is the most lethal gynecological cancer due to high rates of relapse and acquired platinum resistance after conventional chemotherapy [1]. Since sensitivity to platinum determines the prognosis of patients with ovarian cancer, it is important to investigate the factors associated with this condition. The identification and differentiation of ovarian cancer patients in terms of their response to platinum-based treatment is central to advancing ovarian cancer management and has been the subject of intense research. Germline or somatic mutations in the *BRCA1* or *BRCA2* gene have prognostic value in ovarian cancer since ovarian cancer patients with *BRCA1/2* mutations are reported to have a better response to platinum-based treatment [2] and subsequently a longer survival duration than patients without such mutations [3-5]. Although *BRCA1/2* mutations have been found in 11%-20.3% of patients in several studies [6-8], the overall chemosensitivity rates are approximately 70% [9], suggesting that there are other mutations associated with platinum sensitivity [10].

In our previous study, we reported that approximately 10.4% of a disintegrin and metalloproteinase with thrombospondin motifs (ADAMTS) family genes were mutated in patients with high-grade serous ovarian carcinoma. Patients harboring these mutations had significantly longer overall survival (OS), progression-free survival and platinum-free survival independent of *BRCA1* or *BRCA2* mutation, stage, residual tumor, or age, according to the results of a computational analysis of the whole-exome sequencing data from 512 patients in the Cancer Genome Atlas [11]. This finding suggests that ADAMTS mutations partially account for BRCAness [12]. In that study, mutations of ADAMTS 1, 6, 8, 9, 15, 16, 18, and L1 were detected. *ADAMTS16* was one of the most commonly mutated genes.

ADAMTS protease is a secreted extracellular peptidase that mainly consists of three basic structures: a pro-domain, catalytic domain, and ancillary domain [13]. The ADAMTS family has 19 members, and the ADAMTS-like glycoproteins (ADAMTSL) family is often considered the same family because they share similar structures [14-16]. Since the first member of the ADAMTS family was identified in 1997 [17], studies have revealed that the family plays important roles in extracellular matrix development, maintenance, degradation, angiogenesis [13], microfibril biogenesis [16], von Willebrand factor maturation [18], and embryogenesis [19]. The ADAMTS family has been linked to various clinical diseases, including arthritis [20, 21] and thrombotic thrombocytopenic purpura [22, 23]. There is also an increasing number of studies demonstrating the important roles the ADAMTS family plays in the pathogenesis of cancer [24-27]. Mutations of ADAMTS family genes have been detected in various types of cancers, including colorectal, breast, esophageal, and pancreatic cancers and glioblastoma (2, 28-36).

However, the functional role of these mutations in ovarian cancer cells is largely unknown. In this study, we hypothesized that *ADAMTS16* mutations sensitize ovarian cancer cells to platinum-based chemotherapy. To test this hypothesis, we performed *in vitro* studies to compare the drug response in ovarian cancer cells with and without one of the six *ADAMTS16* missense mutations. We also used a well-characterized *in vivo* mouse model to evaluate the response of ovarian cancer cells with *ADAMTS16* mutations to platinum-based therapy.

## RESULTS

### Generation of *ADAMTS16* mutant ovarian cancer stable cell lines

**Figure 1:**
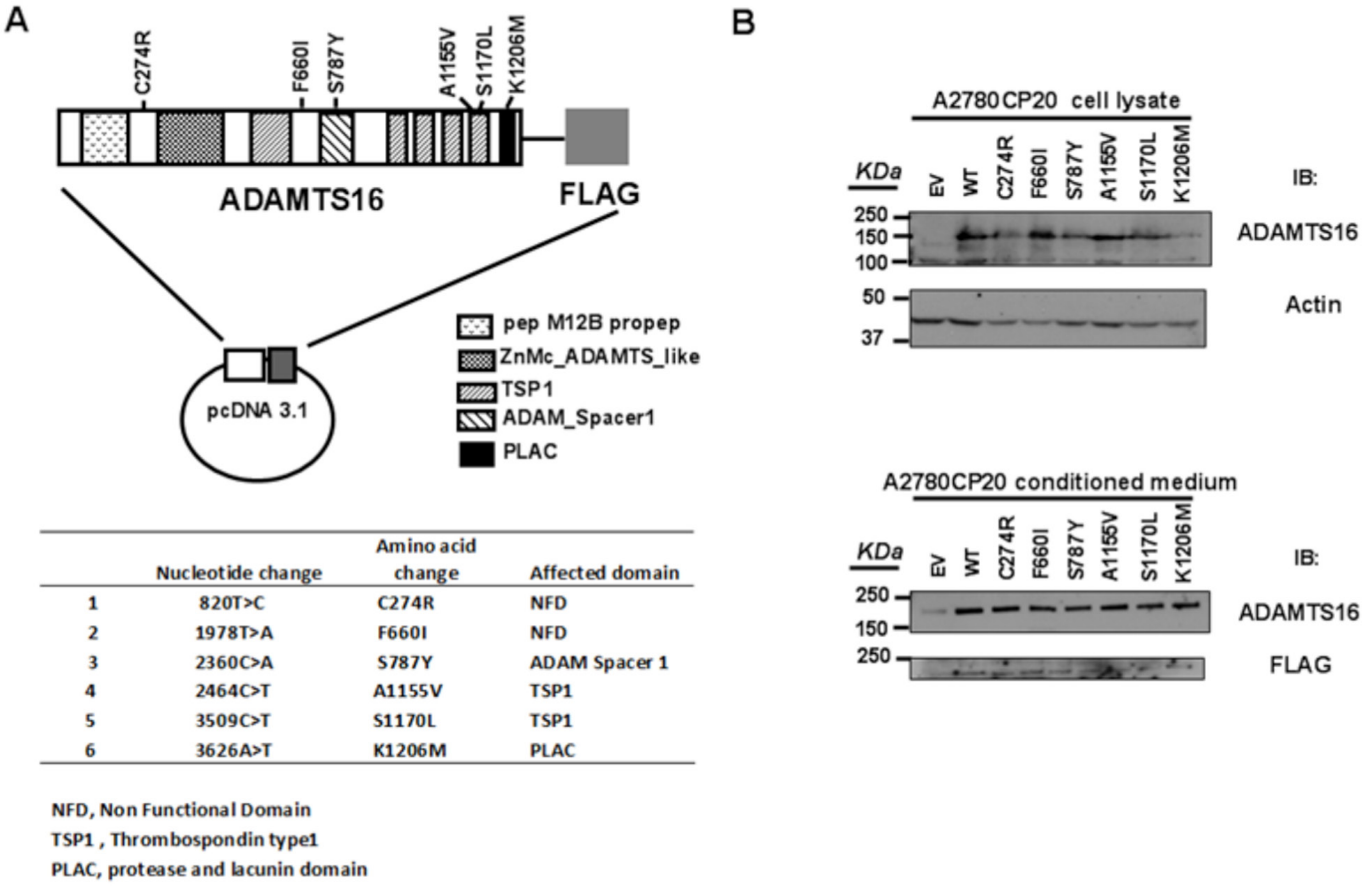
ADAMTS16 mutations and the creation of stable cell lines Panel **A.** shows the vector map, and panel B shows the immunoblotting of transiently transfected A2780CP20 cells. Each vector had wild-type (WT) *ADAMTS16* or a mutant *ADAMTS16* construct (C274R, F660I, S787Y, A1155V, S1170L, or K1206M). After cells had been transfected with vectors, including empty vector, they were selected with neomycin for 2 weeks. Panel **B.** shows ADAMTS16 protein expression in both the cell lysate and conditioned medium, confirmed via immunoblotting using anti-*ADAMTS16* antibody.

With no prior knowledge of the biological function of *ADAMTS16* mutations in ovarian cancer cells, we first generated six *ADAMTS16* mutants on the basis of the missense mutations detected on this gene [11]: C274R, F660I, S787Y, A1155V, S1170L and K1206 (Figure 1A). A1155V and S1170L are in one of the Thrombospondin 1 domains, S787Y is in the ADAM Spacer 1 domain, K1206M is in the protease and lacunin domain (PLAC) and C274R and F660I are in non-functional designated domains. To determine the role of these mutations in ovarian cancer cells, we established ovarian cancer cell lines that stably expressed empty vector (EV) or WT or each of the six ADAMTS16 missense mutants. ADAMTS16 protein level of stable cell lines was detected in both the cell lysate and conditioned medium (Figure 1B) as previously reported in esophageal squamous cell carcinoma [33].

### ADAMTS16 missense mutations inhibit cell growth

**Figure 2:**
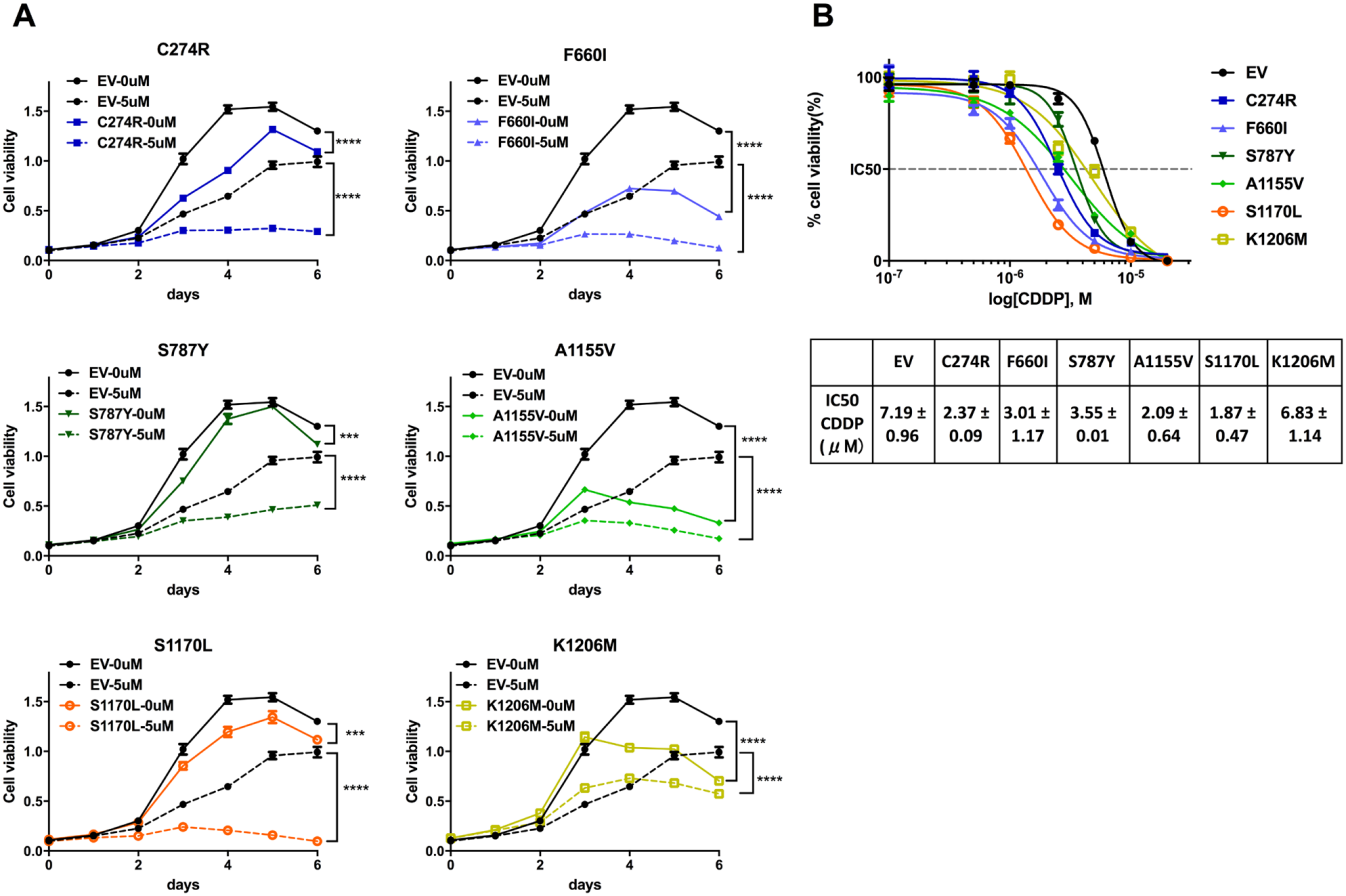
ADAMTS16 mutation improved the response to cisplatin Panel **A.** shows the viability of A2780CP20 cells expressing empty vector (EV) or one of six *ADAMTS16* missense mutations (C274R, F660I, S787Y, A1155V, S1170L, or K1206M) with no treatment or 5 μM cisplatin. Panel **B.** shows the dose-response curves obtained by plotting the viability of EV and all six mutations in seven different cisplatin concentrations (0.1 μM, 0.5 μM, 1.0 μM, 2.5 μM, 5.0 μM, 10 μM, and 20 μM), normalized to the control in a semi-log scale. The cellular 50% inhibitory concentrations (IC50s) of cisplatin for each cell line 5 days after treatment were 7.19±0.48, 2.37±0.09, 3.01±1.17, 3.55±0.01, 2.09±0.64, 1.87±0.47, and 6.83±1.14, respectively (average and SEM), for C274R, F660I, S787Y, A1155V, S1170L, and K1206M. Each average and SEM was obtained from two independent experiments. The IC50s of C274R, F660I, S787Y, A1155V, and S1170L were significantly reduced compared to that of EV (p=0.026, p=0.0144, p=0.0071, p=0.0034, and p=0.0023, respectively; two-tailed unpaired t test).

To determine whether *ADAMTS16* mutations affect cell viability, we first analyzed ovarian cancer cells that stably expressed EV, or one of six *ADAMTS16* missense mutants (C274R, F660I, S787Y, A1155V, S1170L, or K1206M) using a 3-(4,5-dimethylthiazol-2-yl)-2,5-diphenyltetrazolium bromide (MTT) assay over a period of 6 days (Figure 2A solid lines). All six stable *ADAMTS16* mutant cell lines showed significantly decreased viability compared to cells transfected with EV (p=0.0002, p<0.0001, p=0.0017, p<0.0001, p=0.0011, and p<0.0001, respectively, for C274R, F660I, S787Y, A1155V, S1170L, and K1206M).

### *ADAMTS16* missense mutation sensitizes ovarian cancer cells to cisplatin

**Figure 3:**
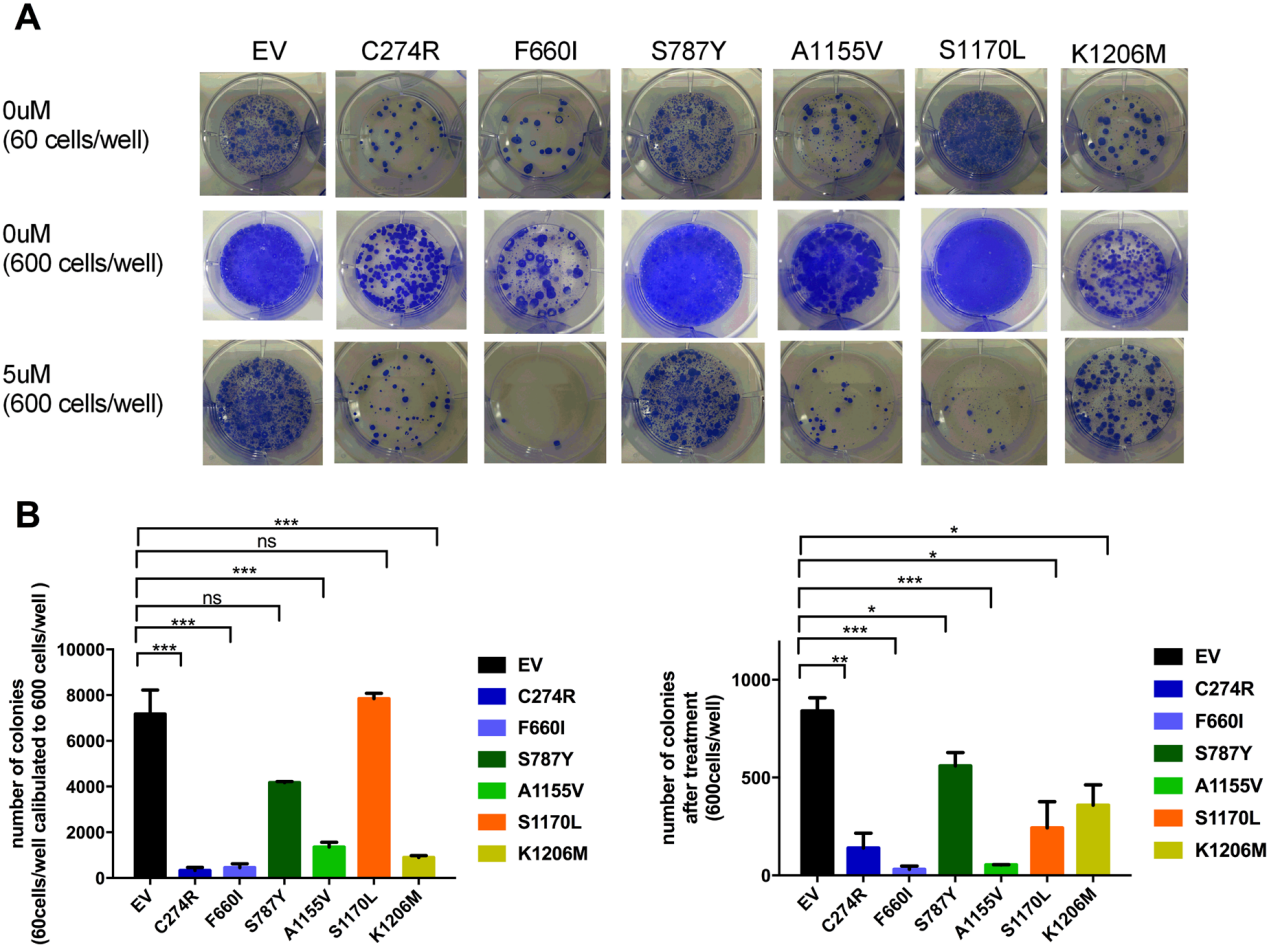
The effect of ADAMTS16 mutations on cancer cells was also observed over time Panel **A.** shows images from the colony formation assay. The first row includes the empty vector (EV) and six mutant cells, seeded at 60 cells/well and incubated for 2 weeks without cisplatin treatment. The second and third rows are the EV and six mutant cells, seeded at 600 cells/well and incubated for 2 weeks without cisplatin treatment and with 5 μM cisplatin. In panel **B.** the bar graph shows the colony number of each cell type after 14 days of incubation without cisplatin treatment (left) and with 5 μM cisplatin (right). As shown in the left graph, we calculated the number obtained from cells seeded at 60 cells/well to 10 times because some cells that were seeded at 600 cells/well showed overgrowth without cisplatin treatment. Panel B shows the average number of colonies without treatment (left) and after 5 μM cisplatin treatment (right). Each bar represents EV, C274R, F660I, S787Y, A1155V, S1170L, and K1206M, respectively. C274R, F660I, S787Y, A1155V, S1170L, and K1206M are respectively compared with EV by the two-tailed unpaired t test (p=0.0151, p=0.0160, p>0.05, p=0.0237, p>0.05, and p=0.0191, left : p=0.0023, p=0.0003, p=0.0423, p=0.0003, p=0.0162, and p=0.0177, right).

Next, we determined whether *ADAMTS16* mutation increases sensitivity to cisplatin. After treatment with 5.0 μM cisplatin, all six stable ADAMTS16 mutant cell lines showed significantly decreased viability compared to those treated with EV control (p<0.0001) (Figure 2A, dotted lines). Next we calculated the 50% inhibitory concentration (IC50) of cisplatin in the EV control and all six stable *ADAMTS16* mutant cell lines by plotting their normalized viability at seven different concentrations (0.1 μM, 0.5 μM, 1.0 μM, 2.5 μM, 5.0 μM, 10 μM or 20 μM) in a semi-log scale. The IC50s were 7.19±0.48, 2.37±0.09, 3.01±1.17, 3.55±0.01, 2.09±0.64, 1.87±0.47, and 6.83±1.14, respectively (Figure 2B, mean and SEM). The IC50s of C274R, F660I, S787Y, A1155V, and S1170L were significantly lower than that of EV (p=0.026, p=0.0144, p=0.0071, p=0.0034, and p=0.0023, respectively). To further assess the effect of a longer incubation period of the mutations on cells with and without cisplatin treatment, we performed a colony formation assay. Without cisplatin treatment, C274R, F660I, A1155V, and K1206M cells formed significantly fewer colonies than did EV control after 14 days incubation (p=0.0151, p=0.0160, p=0.0237, and p=0.0191, respectively) (Figures 3A and 3B). Fourteen days after cisplatin treatment, all six stable *ADAMTS16* mutant cells formed significantly fewer colonies than did the EV control (p=0.0023, p=0.0003, p=0.0423, p=0.0003, p=0.0162, and p=0.0177) (Figure 3B).

### Ovarian cancer cells overexpressing WT *ADAMTS16* did not affect cell growth but made cells resistant to cisplatin

**Figure 4:**
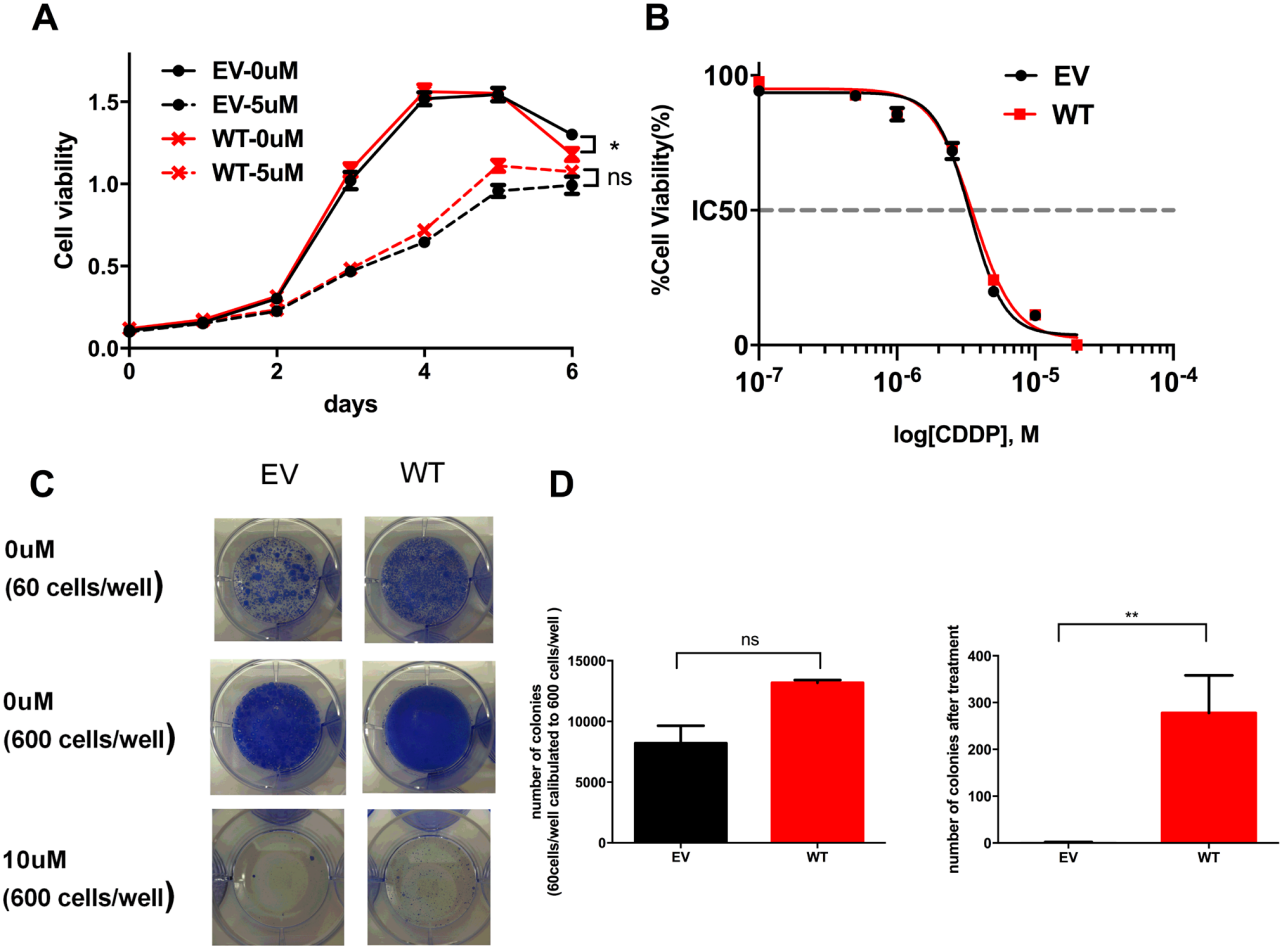
The effect of wild-type (WT) ADAMTS16 on cancer cells Panel **A.** shows the viability of A2780CP20 cells expressing empty vector (EV) and WT *ADAMTS16* with no treatment or 5 μM cisplatin treatment. Panel **B.** shows the dose-response curves obtained by plotting the viability of EV and WT in seven different cisplatin concentrations (0.1 μM, 0.5 μM, 1.0 μM, 2.5 μM, 5.0 μM, 10 μM, and 20 μM), normalized to the control in a semi-log scale. The cellular 50% inhibitory concentrations (IC50s) of cisplatin for each cell line 5 days after treatment were 7.19±0.48 and 8.92±1.88 (average and SEM). Each average and SEM was obtained from two independent experiments. There were no significant differences (p=0.2738; two-tailed unpaired t test). Panel **C.** shows images of the colony formation assay. The first row shows EV and WT cells, seeded at 60 cells/well and incubated for 2 weeks without cisplatin treatment. The second and third rows show the EV and six mutant cells, seeded at 600 cells/well and incubated for 2 weeks without cisplatin or with 10 μM cisplatin. In panel **D.** the bar graph shows the quantification of the colony number of each cell type after 14 days of incubation without cisplatin treatment (left) or with 10 μM cisplatin treatment (right p=0.0040; two-tailed unpaired t test). As shown in the left graph, we calculated the number obtained from cells seeded at 60 cells/well to 10 times because some cells seeded at 600 cells/well showed overgrowth without cisplatin treatment.

To determine whether cells that over-express WT *ADAMTS16* have a proliferative phenotype or are resistant to cisplatin, we used stable *WT ADAMTS16* cells. These cells showed slightly less viability than did the EV control without treatment (p=0.0248), but there was no significant difference between the EV control and WT cells in the cell growth and IC50 (Figures 4A and 4B). In the colony formation assay, there was no significance difference in the number of colonies between the EV control and WT cells after 14 days of incubation, and WT cells formed significantly more colonies than did the EV control at 14 days after treatment with 10μM cisplatin (p=0.0040) (Figures 4C and 4D).

### *ADAMTS16* mutations significantly inhibit cell migration and invasion compared to WT

**Figure 5:**
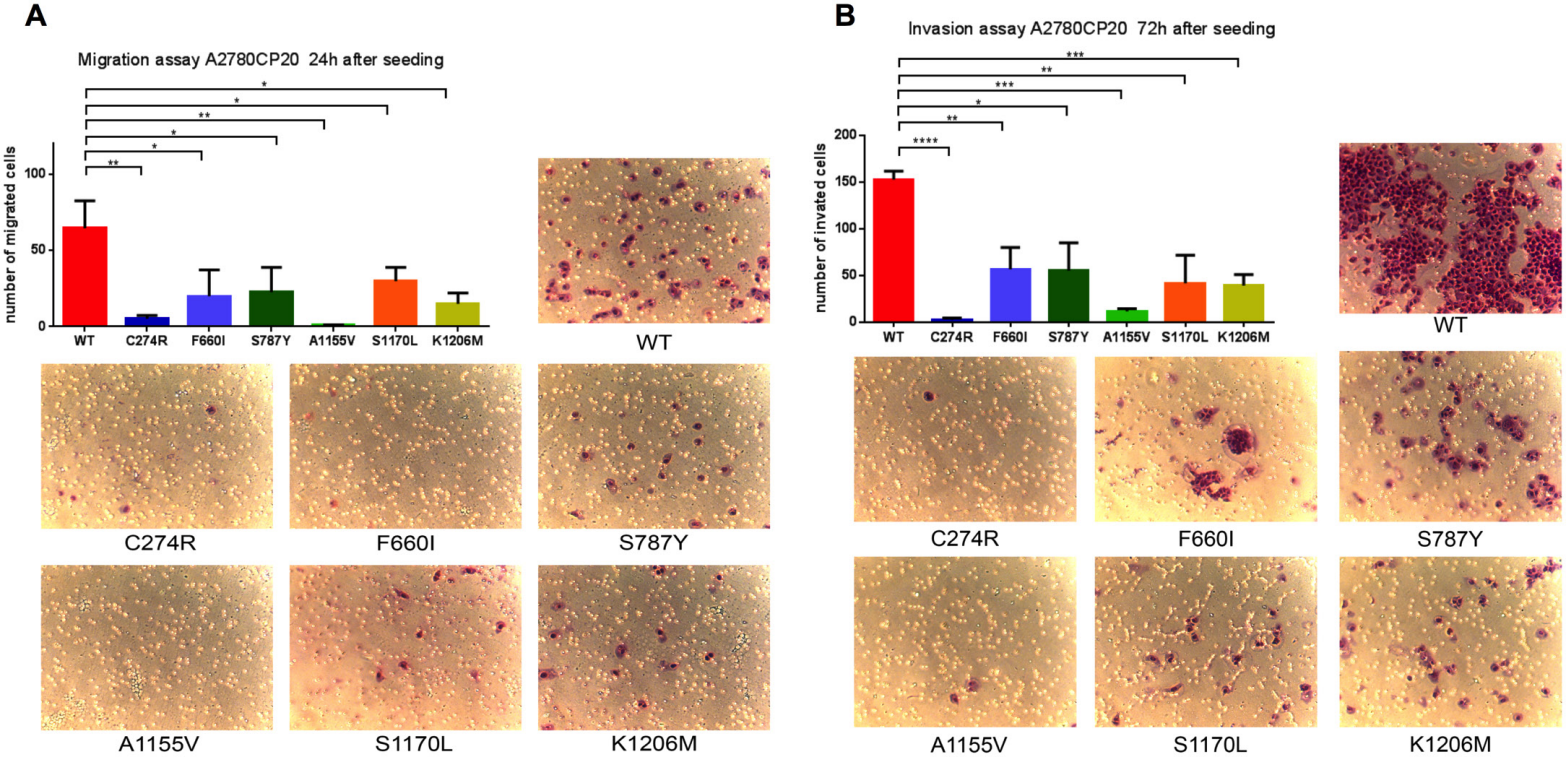
Functional analysis of ADAMTS16 mutations by migration assay and invasion assay Panel **A.** shows the number of migrated cells. Data are presented as the means (±SEM) from triplicate wells. The number of migrated cells in C274R (p=0.0047; two-tailed unpaired t test), F660I (p=0.0360), S787Y (p=0.0395), A1155V (p=0.0036), S1170L (p=0.0401), and K1206M (p=0.0112) cells was significantly lower than that in cells expressing wild-type (WT). Panel **B.** shows representative images from the migration assay. Panel C quantifies the number of invaded cells. Data are presented as the mean (±SEM) from triplicate wells. The number of invaded cells in C274R (p<0.0001; two-tailed unpaired *t*-test), F660I (p=0.0064), S787Y (p=0.0107), A1155V (p=0.0003), S1170L (p=0.0036), and K1206M (p=0.0002) cells was significantly lower than that in cells expressing WT. Panel D shows representative images of the invasion assay.

A previous study reported that deletion of *ADAMTS16* in esophageal cancer cells inhibited invasion [33]. To determine whether the presence of *ADAMTS16* mutation in ovarian cancer cells also changes the phenotype, we performed a migration and invasion assay. Compared to WT ADAMTS16 cells, all six stable ADAMTS16 mutant cell lines showed significantly less migration (p=0.0047, p=0.0360, p=0.0395, p=0.0036, p=0.0401, p=0.0112, respectively, for C274R, F660I, S787Y, A1155V, S1170L, and K1206M) and invasion (p<0.0001, p=0.0064, p=0.0107, p=0.0003, p=0.0036, p=0.0002, respectively, for C274R, F660I, S787Y, A1155V, S1170L, and K1206M)(Figure 5).

### Mutated ADAMTS16 cells had a better response to cisplatin in the mouse model

**Figure 6:**
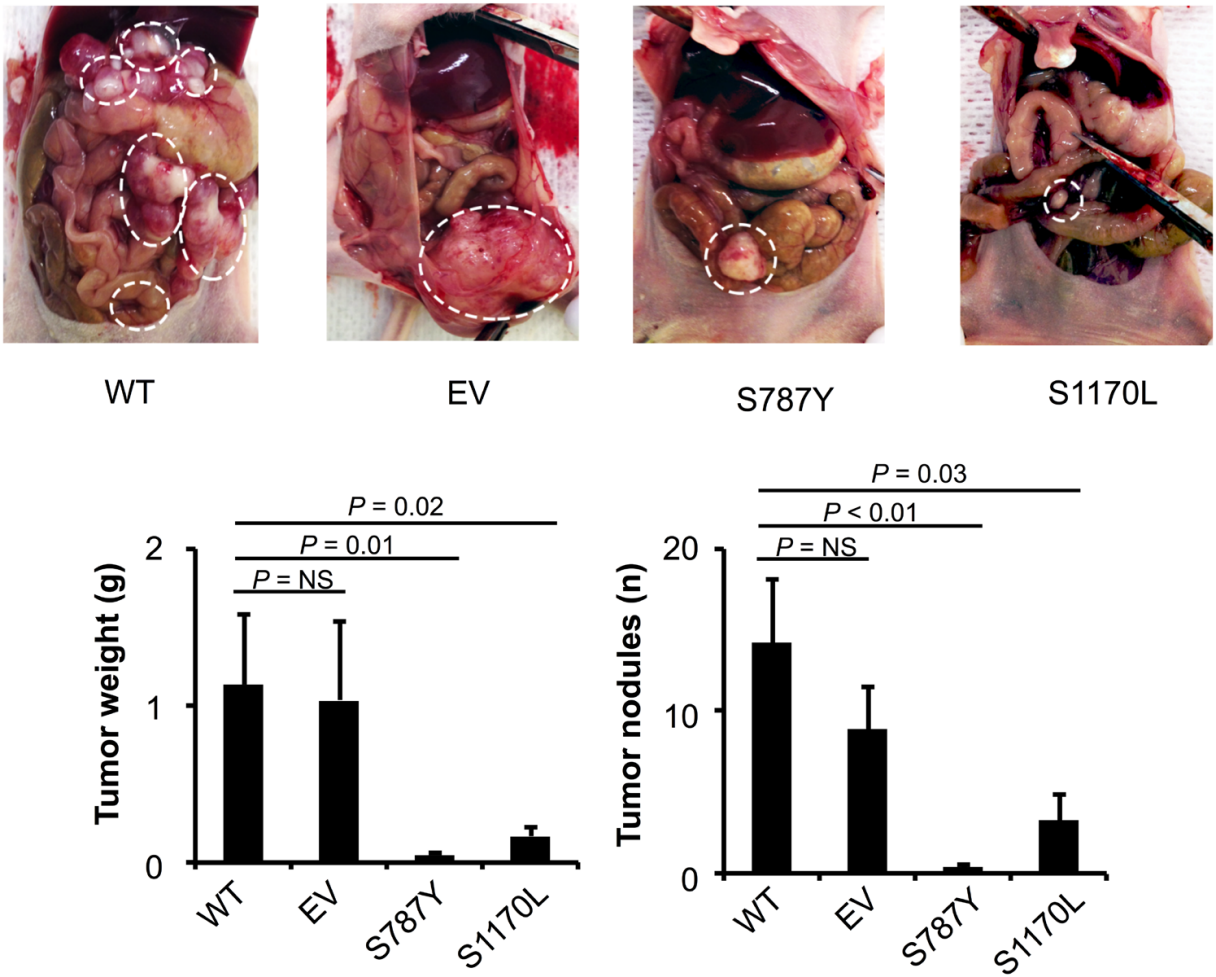
The effect of ADAMTS16 mutation in a mouse model Panel A. show the extent of metastatic spread in treated mice; metastatic areas are outlined with dotted white lines. Nude mice were injected with either A2780-CP wild-type (WT), empty vector (EV), or mutated *ADAMTS16* (S787Y or S1170L) cells. All mice received intraperitoneal cisplatin (160 µg once per week). The tumor weight and number of tumor nodules were compared in the four treated groups (n=7 per group). The results represent the average of the seven treated mice in each group, with error bars representing the SEM. *p<0.05, **p<0.01, comparison with WT by the Mann-Whitney *U* test (Panel B).

Next, we determined the effect of *ADAMTS16* mutations on response to cisplatin *in vivo* using an orthotopic mouse model. The experiment was performed with four groups (7 mice/group), WT A2780-CP20, A2780-CP20 transfected with EV, and A2780-CP20 with *ADAMTS16* mutation at S787Y or S1170L. All the mice received intraperitoneal cisplatin (160µg in 200µl/mouse) once per week, starting 1 week after cell injection. They were all killed when mice from any group became moribund (Figure 6A). Compared to the WT controls, the EV group had no effect on response to cisplatin, however, both of the mutated *ADAMTS16* (S787Y and S1170L) cell lines had a significantly better response to cisplatin (p<0.05 and p<0.01, respectively), as indicated by a reduction in tumor weight or the number of tumor nodules (Figure 6B).

## DISCUSSION

In our previous study, we demonstrated that mutations of eight members of the ADAMTS family (including *ADAMTS16* gene) were significantly associated with chemotherapy sensitivity and longer survival in patients with ovarian cancer, independent of *BRCA1* or *BRCA2* mutations [11]. The identification of the effect of ADAMTS mutations has important implications for clinical prediction. However, unlike that of *BRCA1/2* mutations, the functional role of these ADAMTS mutations in ovarian cancer cells is largely unknown.

To fill in this knowledge gap, we systematically carried out multiple *in vitro* and *in vivo* experimental assays and demonstrated that the introduction of *ADAMTS16* mutants into ovarian cancer cells resulted in both improved sensitivity to cisplatin treatment and reduced cell migration and invasion, providing experimental evidence to support the genomic observation in a large population of patient cohort. The findings of this study, together with those from bioinformatics analyses, offer a cohesive view of the relationship between ADAMTS mutations and chemotherapy response in ovarian cancer patients, and may lead to the identification of novel targets of therapeutic intervention in patients with ovarian cancer. To the best of our knowledge, this is the first report revealing the functional effect of *ADAMTS16* mutations in ovarian cancer cells.

In general, *ADAMTS16* mRNA is highly expressed in the adult brain and ovaries [37]. Although studies have demonstrated an association between *ADAMTS16* and disease such as hypertension [38, 39], osteoarthritis [40], premature ovarian failure [41] and Dupuytren’s disease [42], less is known about the function of the gene than about other members of the ADAMTS family [30]. Few studies have examined the role of *ADAMTS16* in the pathogenesis of cancer. Sakamoto et al showed that ADAMTS16 mRNA was upregulated in esophageal squamous cell carcinoma, and cell growth and invasion were inhibited upon depletion of ADAMTS16 [33]. Castellana et al reported that mRNA expression of *ADAMTS16* was upregulated in invasive ductal carcinoma compared to in ductal carcinoma in situ [30]. On the other hand, a study reported that *ADAMTS16* overexpression resulted in significantly reduced proliferation in chondrosarcoma cells [40]. It is possible that *ADAMTS16* has a different effect on malignancies of epithelial origin and mesenchymal origin, like matrix metalloproteinase 1 [43].

In our *in vitro* study, we observed that cells that expressed mutant *ADAMTS16* C274R, F660I, A1155V, and K1206M had significantly suppressed proliferation without treatment. On the other hand, cells that expressed mutant *ADAMTS16* S787Y and S1170Y had almost no proliferation effects without treatment but showed substantial inhibition after treatment. The latter finding was supported by the result of our mouse model. The results of our study suggests that each missense mutation leads to different cellular changes that affect the response to cisplatin. All six stable *ADAMTS16* mutant cells also showed significantly less migration and invasion than those of WT. This finding supports those of prior studies investigating the role of *ADAMTS16* in tumor invasion [30, 33]. *ADAMTS16* is known to degenerate extracellular protein [37]. Therefore, we think that decrease in cell invasion and migration likely results from the decreased ability of *ADAMTS16* to degenerate extracellular proteins without alternating their gene expression.

Our study has limitations. We evaluated phenotypes of ovarian cancer cells with ADAMTS16 mutations. However, the exact molecular mechanisms of how these mutations sensitize tumor cells to platinum remain unclear because the original function of ADAMTS16 has not been revealed yet. We assume that once ADAMTS16 gene is mutated, the secreted protein reduces its activity to denature extracellular protein. It is possible that this altered enzymatic function plays an important role in impairing tumor microenvironment favorable for cancer cells, thus platinum-sensitivity and migration/invasion are both altered in these cancer cells. Our future investigation to reveal this mechanism will surely bring new insight of chemotherapy in recurrent ovarian cancer patients because therapy approaching to tumor microenvironment would be crucial to cancer cells and administering drug against ADAMTS16 is safer than drugs targeting whole matrix metalloproteinases.

Second, we only used A2780-CP20 in our functional study, because most available ovarian cancer cell lines are relatively sensitive to cisplatin. Therefore, we are limited to cell line selection since we are investigating a molecular event that make resistant cells to sensitivity to chemotherapy. We believe that this weakness was compensated by the fact that we have evaluated six different mutations therefore greatly reducing the possibility that the observation is accidental.

In summary, our investigations revealed that exogenously expressed *ADAMTS16* missense mutations lead to cellular changes that enhance cisplatin sensitivity or inhibit cell growth and suppress tumor invasion and migration in platinum-resistant ovarian cancer. ADAMTS16 is a potential therapeutic target in patients with platinum-resistant ovarian cancer. Further evaluations are needed to reveal the detailed effects of *ADAMTS16* mutation in this disease.

## MATERIALS AND METHODS

### Cell culture

A2780CP20 cells, platinum resistant human epithelial ovarian cancer cells that are derived from A2780 platinum-sensitive cells, are obtained from Dr. Anil K. Sood [44]. Cells were cultured at 37°C in 5% CO_2_ in RPIM1640 (Cellgro) supplemented with 10% fetal bovine serum (Hyclone) [45, 46].

### ADAMTS16 expression plasmid

Full-length wild-type (WT) human *ADAMTS16* cDNA, kindly provided by Dr. Ian M. Clark (University of East Anglia) [40], was cloned into pcDNA3.1 vector (Invitrogen) with a c-terminal Flag tag (Figure 1A). The six missense mutations of *ADAMTS16* were introduced using the QuikChange MultiSite-Directed mutagenesis kit (Agilent Technologies) according to the manufacturer’s protocols. All mutations were confirmed by DNA sequencing, as previously described [47].

### Transfection and generation of stable cell lines

A2780CP20 cells were transfected in six-well plates with WT or one of six different mutant *ADAMTS16* expression plasmids using FuGENE HD transfection reagent (Roche Applied Science) according to the manufacturer’s instructions. After 24 hours, cells were tripsinized and seeded in 10-cm dishes, and the remaining cells were used to confirm transfection by Western blot analysis. Cells were cultured in G418 (Invitrogen)- containing media for 2 weeks. After the selection, the mixtures of stable cells were confirmed by checking the cell lysate and the conditioned media by Western blot analysis [47].

### Validation of stable cells by western blot analysis

Cells were seeded in six-well plates in serum-containing media for 24 hours. After 24 hours of culture in serum-free media, conditioned media were harvested, centrifuged, and stored at -20°C. Whole Cell lysates were harvested and lysed using RIPA buffer with a protease and phosphatase inhibitors cocktail (Thermo Scientific). Samples (conditioned media and whole cell lysate) were separated on 7% polyacrylamide gels and transferred to nitrocellulose membranes. After blocking in PBS containing 5% nonfat-milk, membranes were incubated overnight at 4°C with anti-*ADAMTS16* and Anti-Actin (Santa Cruz) antibodies and for 1 hour at ambient temperature with secondary antibodies (Santa Cruz). The proteins were visualized using the chemiluminescent substrate (Rockford, IL).

### Cell viability assay

Cells were seeded at 3000 cells/well in a 96-well plate in quadruplicate. After 12 hours, they were treated with 0 μM, 5 μM, or 10 μM cisplatin. Cell viability was measured at 0 h as day 0 and every day for 6 days after treatment with 0.5 mg/ml MTT reagent in PBS (Sigma-Aldrich) at 590 nm using the Tecan SpectraFluor microplate Reader and Magellan 6 software (Tecan Group, Ltd.). For the dose dependent curve, cells were treated with 0 μM, 0.5 μM,1 μM, 2.5 μM, 5 μM, 10 μM, or 20 μM cisplatin. Cell viability was measured 5 days after drug treatment [45, 46].

### Colony formation assay

Cells were seeded in six-well plates at 60 and 600 cells/well. After 12 hours, they were treated with 0 μM, 5 μM, or 10 μM cisplatin. The plates were incubated for 2 weeks and the medium was changed twice per week. Colonies consisting of more than 50 cells were counted under a microscope after staining.

### Cell migration and invasion assays

The cell migration and invasion assay was performed in duplicate using Matrigel-coated transwell chambers. The cells were plated in 500 μl of serum-free medium and allowed to migrate or invade towards a 10% FBS medium for 24 h or 72 h. Cells that invaded into the underside of the filter were fixed and stained with Hema-Diff solution (Fisher). The numbers of invaded cells from 5 randomly chosen fields were counted for each membrane, as previously described [48].

### Mouse model

Female athymic nude mice were purchased from Taconic Farms and maintained in pathogen-free conditions. They were cared for according to the guidelines of the American Association for Accreditation of Laboratory Animal Care and the U.S. Public Health Service Policy on Human Care and Use of Laboratory Animals. All *in vivo* experiments and protocols were approved by MD Anderson’s Institutional Animal Care and Use Committee. The development and characterization of the orthotopic mouse models of epithelial ovarian cancer have been described previously [49, 50] The experiment was performed with four groups (seven mice/group): A) WT A2780-CP20 (WT), B) A2780-CP20 transfected with empty vector (EV), and C and D) A2780-CP20 with ADAMTS mutations (S787Y and S1170L). These two mutations were selected for *in vivo* model experiments because they did not exhibit significant inhibition on cell growth compared to the empty vector (Figure 2A and 4A). For the cell injections, cells were trypsinized at 60%-80% confluence and centrifuged at 1200 RPM for 6 min at 4°C. They were then washed twice with phosphate-buffered saline (PBS) and reconstituted in Hanks balanced salt solution (HBSS) to a desired concentration (5.0 X 10^6^ cells/mL). Two hundred microliters of the cell suspension containing 1.0 X 10^6^ cells were injected into the peritoneal cavity of each mouse. The treatment was started 1 week after injecting the cells. All the mice in all four groups received intraperitoneal cisplatin treatment (160µg in 200µl/mouse) once per week. All the mice were killed when mice from any group became moribund. Tumor weight and the number of tumor nodules were recorded.

### Statistical analysis

Each experiment was repeated two times. All data are represented as mean ± SEM. Statistical analyses were performed using GraphPad Prism6 Software (Graph Pad).
